# African Trypanosomiasis in Travelers Returning to the United Kingdom

**DOI:** 10.3201/eid0801.010130

**Published:** 2002-01

**Authors:** David A.J. Moore, Mark Edwards, Rod Escombe, Dan Agranoff, J. Wendi Bailey, S. Bertel Squire, Peter L. Chiodini

**Affiliations:** * Hospital for Tropical Diseases, London, United Kingdom †Liverpool School of Tropical Medicine, Liverpool, United Kingdom

**Keywords:** trypanosomiasis, African, Trypanosoma brucei, therapy, travel

## Abstract

Two returning safari tourists with African trypanosomiasis were admitted to the Hospital for Tropical Diseases, London, in a 3-day period, compared with six cases in the previous 14 years. We describe the clinical features, diagnosis, and problems encountered in accessing appropriate therapy, and discuss the potential for emergence of this disease in increasingly adventurous international travelers.

A 51-year-old man returned from a 14-day game-viewing vacation in the Luangwa Valley of southern Zambia on October 11, 2000. He had been well while traveling but had sustained numerous mosquito and tsetse fly bites. Two days after his return, he noticed an enlarging, slightly tender, erythematous lesion on his right shoulder (Figure 1a). Two days later he became very ill with severe generalized myalgia, abdominal discomfort, diarrhea, vomiting, headache, fever, rigors, and sweats, but did not seek medical attention. On day 10 after his return, he consulted his primary-care physician and was admitted to his local hospital. No malaria parasites were seen on a blood film, but numerous trypomastigotes of *Trypanosoma* sp. were identified, confirming the diagnosis of African trypanosomiasis. The patient was transferred to the Hospital for Tropical Diseases, London. The trypomastigotes of *T. b. rhodesiense* are indistinguishable morphologically from those of *T. b. gambiense*, but from the epidemiology of these infections the patient was presumed to have *T. b. rhodesiense* infection, for which the recommended initial therapy is intravenous suramin.

**Figure 1:**
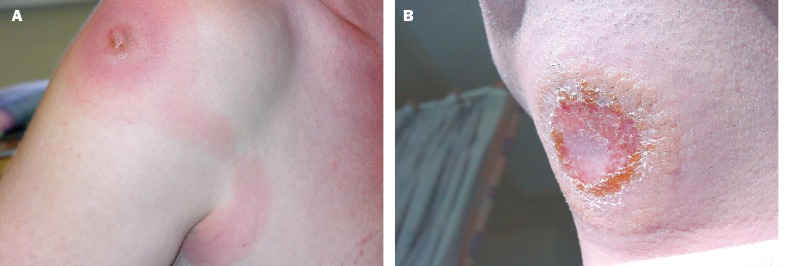
**a.** Trypanosomal chancre on shoulder of patient 1, with lymphangitis toward axilla. **1b.** Trypanosomal chancre on throat of patient 2.

Two days later the hospital admitted a second returning traveler with *T. brucei* (also presumed to be *rhodesiense*) infection, in this case acquired during a 14-day safari vacation to Kenya and Tanzania. This 30-year-old male patient visited the Ngorongoro Crater, Serengeti National Park, and Lake Manyara (Figure 2), but noted tsetse bites only while in the Serengeti, 7 days before symptom onset. He reported a 2-day history of an enlarging, painless, nonpruritic skin lesion on his neck, intermittent fever, a single rigor, and two brief episodes of diarrhea and vomiting. A characteristic trypanosomal chancre was present on the skin of the submandibular region (Figure 1b). The second patient also received intravenous suramin.

**Figure 2:**
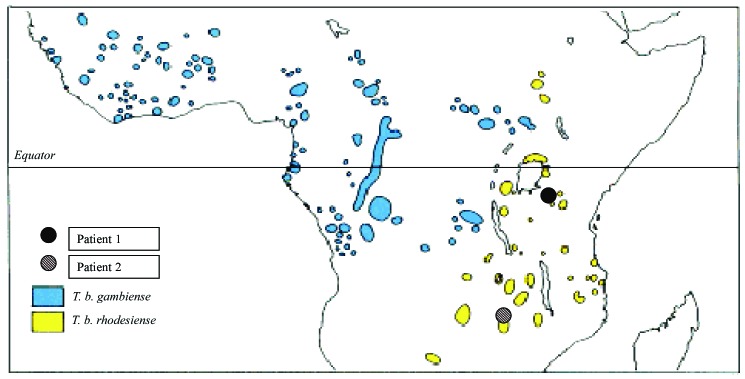
Map of sub-Saharan Africa, indicating itineraries of two patients with *Trypanosoma brucei rhodesiense* infection

Both patients exhibited a rapidly rising systemic inflammatory response (initial C-reactive protein values 235 and 185 mg/L, respectively; normal <5 mg/L), lymphopenia (nadir counts 0.1 and 0.42 x 10^9^/L; normal range [NR] 1.5-4.0 x 10^9^/L), thrombocytopenia (nadir counts 30 and 119 x 10^9^/L; NR 150-400 x 10^9^/L), and mild hyponatremia (129 and 131 mmol/L; NR 136-145 mmol/L). Neither patient had notable coagulopathy. Specific antitrypanosomal serologic testing by immunofluorescence assay (IFA) yielded the following results: patient 1: day 1 immunoglobulin (Ig) M 1:40, IgG negative; day 28 IgM 1:20, IgG 1:80; and patient 2: day 1 IgM negative, IgG 1:40; day 28 IgM 1:20, IgG 1:160. Screening dilution was 1:20. Specific antitrypanosomal IFA was negative for all cerebrospinal fluid (CSF) samples tested.

Both patients were treated in accordance with a protocol developed and used in Malawi and Uganda by workers from the Liverpool School of Tropical Medicine. In brief, after a test dose to exclude immediate hypersensitivity, intravenous suramin (reconstituted in sterile water to a 10% solution, with the required dose added to 200 mL 5% dextrose and infused over 2 hours) is administered at doses of 5, 10, and 20 mg/kg on days 1, 3, and 5, respectively (maximum 1.5 g/dose). Fresh CSF samples are examined on day 5 to determine whether there is evidence of central nervous system (CNS) involvement, as defined by elevated IgM, pleocytosis, or presence of trypomastigotes. Lumbar puncture is deliberately deferred until circulating trypanosomes have been cleared, to minimize the theoretical risk of iatrogenic introduction of trypomastigotes into the CSF. This delay also aids interpretation of CSF findings as, even if there were blood in the CSF, any trypanosomes found would still indicate CNS infection. CNS involvement is treated with melarsoprol in 12 escalating doses over 4 weeks and, although an accelerated 10-day schedule for *T. b. gambiense* has recently undergone successful clinical trials in Angola (*1*), this regimen has not been validated for the treatment of *T. b. rhodesiense*. Suramin is ineffective in CNS disease; however, melarsoprol is a toxic arsenical compound associated in some cases with encephalopathy, which can be fatal, and is thus best avoided unless definitely required. If there is no evidence of CNS involvement, therapy with suramin (20 mg/kg intravenously, maximum 1.5 g/dose) is continued and administered on days 9, 16, 23, and 30.

Both our patients received the full 7 doses of suramin, as neither had evidence of CNS disease on CSF examination. Interestingly, both patients described a subjective dysesthesia of the fingertips during suramin therapy, which resolved on treatment completion and was not associated with any objective evidence of neuropathy on clinical examination. Transient moderate proteinuria, a known side effect of suramin, was noted in both patients and resolved after treatment.

The number of patients with imported *T. b. rhodesiense* visiting clinicians in nonendemic areas is likely to increase. For *T. b. rhodesiense*, which predominates in East Africa, wild and domestic animals are important reservoirs. Wherever people come into close proximity with these reservoirs (when visiting game parks, for example) the potential exists for transmission to humans by the tsetse fly vectors of the *Glossina morsitans* group. In contrast, for *T. b. gambiense*, which predominates in West Africa, the human population itself is the most important reservoir and *G. palpalis* tsetse flies are the most important vector. The resurgence of sleeping sickness in Africa is mainly due to *T. b. gambiense*. Countries reporting epidemics of *T. b. gambiense* include Sudan, Angola, and the Democratic Republic of Congo, where the number of cases (certainly underreported) increased from 7,700 in 1990 to 27,044 in 1998 (*2*). The areas affected are not usually visited by tourists or adventure travelers. Further epidemiologic information is available on the World Health Organization website (*3*). In large part, the resurgence of *T. b. gambiense* is due to the breakdown of health systems in regions of civil and military unrest and is cause for considerable concern.

Imported African trypanosomiasis has hitherto been rare in the United Kingdom, with only six cases in the last 14 years at the Hospital for Tropical Diseases, most recently in 1996. Aside from the temporal proximity of the two cases described, we were concerned that both patients had acquired infection in areas commonly frequented by international safari-tourists from around the world. We know of nine subsequent additional European cases of imported African trypanosomiasis (*T. b. rhodesiense*) from Tanzania, and of an Australian who acquired the infection from the same location in 1998 (*4*). In 1999 Sinha et al. reported only the 20th and 21st cases of *T. b. rhodesiense* infection imported into the United States (*5*). However, increasing numbers of travelers are exposed to tsetse fly bites in disease-endemic areas, and many of the game animals that the safari-tourist will view, including bushbuck, waterbuck, and lion, are potential reservoirs. Furthermore, with increases in tsetse fly activity reported in many regions of sub-Saharan Africa (including the Luangwa Valley), the potential for further cases is substantial. Neither of the locations from which our patients acquired their infections is novel, but the sudden appearance of two cases in such a short time is noteworthy. Clinicians should thus be alerted to the possibility of an increase in cases of imported African trypanosomiasis in safari-tourists.

Next, we emphasize three key diagnostic points. First, diagnosis in the two cases described was straightforward because, as is often the case with early presentations of *T. b. rhodesiense*, the patients had classic chancres and parasites were easily identified in the blood. Had the patients delayed in seeking medical attention or been exposed to *T. b. gambiense*, identifying parasites in the blood would have been more difficult, leaving the risk of disease progression to neurologic involvement before the diagnosis could be confirmed. Second, when CSF samples are taken, either to confirm or exclude late-stage neurologic trypanosomiasis, they must be examined in the laboratory within 20 minutes of the sample’s being drawn. After this time, the parasites are likely to lyse spontaneously and be missed. Third, serology is not required for the diagnosis of *T. b. rhodesiense* in blood, as the levels of parasitemia are high. Furthermore, the number of *T. b. rhodesiense* clones circulating in East Africa has led to problems with test sensitivity, and the specificity is not well characterized. Serology, however, can aid the decision as to whether there is CNS involvement.

Finally, we emphasize the difficulties that we encountered (on a Saturday) in securing a supply of the therapeutic agent. Suramin was available neither from our own pharmacy (the last batch had been discarded after its expiration date passed 2 years earlier) nor from eight other regional tropical or infectious disease units in the United Kingdom, France, and Belgium. Previous use of suramin in oncology prompted us to contact a large regional oncology unit in London, but the drug was not available. Initial inquiries of hospital pharmacies in Liverpool was not promising. However, a supply was finally located at the Liverpool School of Tropical Medicine, and 3 doses, sufficient for 5 days of treatment, were sent to London by courier. The rest of the drug, obtained from the Centers for Disease Control and Prevention, arrived on day 5 after admission. The Hospital for Tropical Diseases has since secured a supply of suramin from the manufacturer in Germany, which we have in turn provided to another hospital for treatment of another patient.

There is grave concern about the international availability of a number of antiparasitic drugs, with considerable uncertainty about the sustainability of supplies of several, including suramin and melarsoprol, which manufacturers deem commercially unattractive. As the current epidemic of trypanosomiasis continues, access to effective therapies must be ensured in disease-endemic areas. Although drug resistance is potentially a serious problem, ensuring access to the currently available therapies poses a considerable challenge, which ultimately may depend on governmental or World Health Organization intervention. This issue has also been highlighted recently elsewhere (*6*).

A logistical problem in nonendemic areas, where imported trypanosomiasis is likely to remain rare, is access to agents such as suramin, for which the demand is small and which are therefore very seldom stocked by general hospital pharmacies but are needed urgently when a patient is admitted. Fortunately, our first patient was relatively well and not adversely affected by the 8-hour delay in initiation of therapy, but this might not always be the case. It is vital that a mechanism is in place to allow physicians working in nonendemic areas who are involved in the management of patients from trypanosomiasis-endemic areas to have access to suramin and several other essential but rarely used agents (such as melarsoprol, eflornithine, nifurtimox, and benznidazole; the last two drugs used for treatment of South American trypanosomiasis) from a central repository 24 hours a day, 7 days a week.
